# Functional Outcomes of Capitellum Fractures Treated by Open Reduction and Internal Fixation with Herbert Screw: A Descriptive Cross-sectional Study

**DOI:** 10.31729/jnma.5188

**Published:** 2020-10-31

**Authors:** Kapil Mani KC, Parimal Acharya, Suman Babu Marahatta, Arun Sigdel, Amuda KC, Sudip Chandra Dahal

**Affiliations:** 1 Department of Orthopedics, Civil Service Hospital, Minbhawan, Kathmandu, Nepal; 2 Nepalese Army Institute of Health Sciences, Sanobharyang, Kathmandu, Nepal; 3 Department of Statistics, Civil Service Hospital, Minbhawan, Kathmandu, Nepal

**Keywords:** *fracture*, *surgery*, *treatment*

## Abstract

**Introduction:**

Based on the complex intra-articular nature of capitellum fractures, it has been sometimes difficult to formulate a universally accepted method of surgical treatment. The purpose of this study is to present the functional outcomes of capitellum fractures after fixation with Herbert screw including the safety and tips of the surgical approach.

**Methods:**

This descriptive cross-sectional study was done from December 2014 to November 2019. Ethical approval was taken. The study included 22 capitellum fractures treated by open reduction and internal fixation with Herbert screws either lateral or anterolateral approach. Functional outcomes were assessed with Mayo elbow performance index scores at the latest follow-up visit. Convenient sampling was done. Data entry was done using the Statistical Package for the Social Sciences (version16.0).

**Results:**

Out of 22 surgeries, the average time to unite the fracture was 11.13±1.20 weeks (range 9 to 15). The mean range of movement for flexion and extension was 138.41±8.22 degree while the mean supination and pronation range was 161.59±6.79 degree. The average time of follow-up in this series was 37.45±9.43 weeks (range 22 to 58 weeks). Similarly, the mean Mayo elbow performance index score at the latest follow-up was 90.22±8.65 (range 70 to 100).

**Conclusions:**

Careful assessment and radiological evaluation, anatomical reduction, and stable fixation with Herbert screws maintaining the minimal damage to the articular cartilage can maximize the functional outcomes and minimize the incidence of complications.

## INTRODUCTION

Capitellum fractures which result mainly because of axial load transmitted through the elbow represent 1% of whole elbow fractures.^1,2^ It is sometimes not uncommon to miss the undisplaced or minimally displaced capitellum fractures by orthopedic surgeons unless performed true lateral view of the elbow and a high index of suspicion.

Treatment options for these fractures include both conservative and surgical treatment.^3–9^ However, complex capitellum fractures including the metaphyseal comminution and ipsilateral radial head fracture makes optimal surgical approach and treatment debatable.^3–9^ Some studies have used the anterolateral rather than lateral approach to better visualization of fractures.^10–12^ Even though various implants are available for fixation, Herbert screw provides stable fixation, excellent compression at the fracture site, and non-prominence of the implant within the joint.^13^

The purpose of this study is to present the functional outcomes of capitellum fractures after fixation with Herbert screw including the safety and tips of surgical treatment.

## METHODS

This was a descriptive cross-sectional study performed in Civil Service Hospital, Kathmandu, Nepal from December 2014 to November 2019. A total of 27 capitellum fractures were treated surgically during this period, however, 5 patients were lost during the follow-up and finally included 22 patients in this study. Permission for the study was taken from the institutional review board of our hospital (IRC Protocol No: 08/2020) as well as written consent from each patient was taken to participate in the study. All the surgeries were performed by either the corresponding author or second author either independently or jointly. Patients with closed capitellum fractures with or without comminution or extension to trochlear ridge and age range of 20 to 70 years were included in the study. Those with open fractures, more than 10 days after injury, posterior cortex comminution, ligamentous laxity, previous degenerative or inflammatory arthritis, and those not fit for surgery were excluded from the study. Preoperative radiographs of anteroposterior (AP) and lateral views were done in all cases. Those with radiographic features suspected of associated fractures or comminuted capitellum fractures were further assessed by computed tomography (CT) scan including the 3D views. The sample size was calculated as,

$$\begin{array}{ll} \text{n} & \text{= }{\text{Z}}^{\text{2}}\times \text{p}\times \text{(1}-\text{p)}/{\text{e}}^{\text{2}}\\ & \text{= }{\left(1.96\right)}^{\text{2}}\times (0.5)\times (1-0.5)/{\left(0.2\right)}^{\text{2}}\\ & \text{= }22 \end{array}$$

Where,

n = sample size
Z = 1.96 at 95% Confidence Interval (CI)
p = population proportion, 50%
e = margin of error, 20%

Therefore, 22 patients were enrolled in the study.

Capitellum fractures in this study were further classified by the Dubberley classification system. Type 1 fracture is primarily the capitellum fracture with or without involving the trochlear ridge. Type 2 fracture is a single combined fragment involving the capitellum and trochlea whereas in type 3 both capitellum and trochlear fragments are separate. Those fractures without or with involving the posterior cortex are designated as A and B.

Under general anesthesia or peripheral block, the patient was positioned supine on the operation table. A pneumatic tourniquet was applied at the level of midarm and a varus-valgus stress test was performed to rule out the ligamentous injuries before giving the incision on the elbow. Usually, the surgery was done through the lateral approach where around 6 to 8 cm long skin incision was made with 4 to 6 cm proximal and 2 cm distal to joint level. Proximally the intermuscular plane is between the brachioradialis (BR) and extensor carpi radialis longus (ECRL) anteriorly and triceps posteriorly while distally in between the extensor carpi radialis brevis and extensor digitorum communis. Superficial dissection was done and common extensor muscle origin in the lateral column was identified. The forearm is pronated to move the posterior interosseous nerve (PIN) away from the surgical field. The common extensor origin (mainly BR and ECRL) along with anterior capsule was reflected anteriorly while the dissection was extended distally between the extensor carpi radialis brevis (ECRV) and extensor digitorium communis (EDC) to expose the fracture site taking due care to avoid the injury on the anterior aspect of the lateral ulnar collateral ligament (LUCL). The fracture site was visualized clearly by removing the soft tissue, hematoma followed by reduction, and temporarily fixed by K wire from anterior to posterior. Anatomical reduction of capitellum was confirmed by the perfect match of metaphyseal and trochlear ridge with fractured fragments. If the fragments were either multiple or joined with the trochlear fragment, they are reduced separately and fixed with K wires. Depending on the medial extension of the fracture, the anteromedial approach rather than the lateral approach can be used intra-operatively. Now two guide wires (most commonly) were put from anterior to posterior direction followed by cannulated drilling and two Herbert's screws buried completely on articular cartilage. The stability of fixation was assessed by flexion-extension and supination pronation movement. Common extensor origins were repaired back to the lateral column and the wound was closed in two layers. Compressive sterile dressing was applied, and a long arm posterior plaster slab was applied with the elbow kept in flexion of around 90°.

The wound was examined on the third day and dressing was done. Patients were followed up in OPD two weeks, then every six weeks until the union has occurred and every year thereafter. After removing the suture and discontinuing the posterior slab on 2 weeks, gentle passive and active mobilization exercise of the elbow joint was started along with wrist and shoulder mobilization exercises. Strengthening exercises were delayed until clinical and radiographic evidence of bone union was seen. The functional outcome of the elbow was assessed using the Mayo elbow performance index (MEPI) score at the latest follow-up. A radiograph was done at each follow-up to assess the radiological union, signs of avascular necrosis, and osteoarthritis.

Statistical analyses were performed using the SPSS software (version 16.0). Quantitative variables were documented as mean±standard deviation.

## RESULTS

The average age of patients in our study was 35.86±11.18 years (range 20-64) (Table 1). Fourteen (63.63%) were male while 8 (36.37%) were female. There were 14 (63.63%) fractures on the left side and 8 (36.37%) on the right side. The majority of injury was because of RTA 9 (40.90%) followed by fall on the ground 8 (36.37%) and sports-related injury 5 (22.73%). Thirteen (59.09%) fractures were Dubberley type 1A, 6 (27.28%) were type 2A and 3 (13.63%) were type 3A. The mean intraoperative time was 85.68±10.38 minutes (range 70 -115). The average time to unite the fracture was 11.13±1.20 weeks (range 9 to 15). The mean range of movement for flexion and extension was 138.41±8.22 degree while the mean supination and pronation range was 161.59±6.79 degree (Table 2). The average time of follow-up in this series was 37.45±9.43 weeks (range 22 to 58 weeks). Similarly, the mean MEPI score at the latest follow-up was 90.22±8.65 (range 70 to 100)

**Table 1:** Showing demography of study participants (n = 22)

Characteristics (n =22)	Findings n (%)
Age (years)	35.86±11.18 years (range 20-64).
Sex	
Male	14 (63.63%)
Female	8 (36.37%)
Side	
Right	8 (36.37%)
Left	14 (63.63%)
Mechanism of injury	
RTA	9 (40.90%)
Fall on ground	8 (36.37%)
Sports injuries	5 (22.73%).
Classification of fractures (Dubberley)	
1A	13 (59.09%)
2A	6 (27.28%)
3A	3 (13.63%)

**Table 2:** Showing surgical parameters.

Characteristics	Findings
Mean operative time (minute)	85.68±10.38 (range 70-115).
Time to unite the fracture (week)	11.13±1.20 (range 9 to 15).
ROM flexion/ extension (degree)	138.41±8.22 (range 22 to 58 weeks).
ROM pron/ supination (degree)	161.59±6.79
MEPI score	90.22±8.65 (range 70 to 100)
Follow up period (week)	37.45±9.43

## DISCUSSION

The successful management of capitellum fractures depends on early diagnosis with a high index of suspicion, thorough clinical assessment to rule out ligamentous and other bony injuries, proper radiographic work up to evaluate the geometry of fractures, appropriate surgical approach, and stable fixation. Similar to other fractures in the upper limb, capitellum fracture is more common in non-dominant hand and young age group because of the frequent engagement of male patients in outdoor activities, however,the prevalence of fracture is four times higher in female than in the male. It has been attributed to increased carrying angle in a female that impacts more shear force in capitellum getting the fracture along with weaker bone (osteoporosis) in the female.^14,15^ The average age of patients in our study was 35.86±11.18 years (range 20-64). Fourteen (63.63%) were male while 8 (36.37%) were female. There were 14 (63.63%) fractures on the left side and 8 (36.37%) on the right side. Similarly, RTA is the major mode of injury to have the fractures 9 (40.90%) and Dubberley type 1A, 6 (27.28%) is the commonest variety of fractures. The demographic profiles of our study are similar to other studies.^2,3,4,14,15^

Capitellum fracture lies in the coronal plane and it may be missed in anteroposterior view unless subtle clinical findings like swelling and decreased elbow range of motion have been correlated along with proper true lateral views of the elbow joint.^16^ Also, a pathognomonic “double arc sign” formed by the overlap of the subchondral bone of capitellum and trochlear ridge in true lateral view is appreciated in type IV variety of McKee classification.^8^

The type of fracture fixation method is also an interest in the management of capitellum fractures. Different methods like K wires, metallic screws, biodegradable implants have been reported in the literature for fracture fixation.^13^ Out of these, metallic screws are a more favorable option because they provide stable fixation for early range of movement which is vital for good functional outcomes.^17,18^ There are different types of screws available such as cortical, lag, cannulated, cancellous headless, and Herbert screws, however, no direct comparison has been possible because of heterogeneous reporting of clinical outcomes. Nowadays, Herbert screw has been considered as a first choice implant because of its biochemical properties of stable fixation, excellent compression, non-prominence of implants within the intra-articular surface, and no need for removal of implants.^19^

Elbow fractures are associated with ligamentous injuries which may lead to instability of the elbow. Giannicola et al reported elbow dislocation with capitellum fractures which are associated with lateral collateral ligament (LCL) and medial collateral ligament (MCL) injury leading to a potential pattern of complex elbow instability and poor functional outcomes.^20^ However, isolated capitellum fractures may not be commonly associated with ligament injuries. In the current study, no single case of ligament injurywas found during the intraoperative assessment. Considering the significant poor functional outcomes of capitellum fractures associated with ligament injuries, even though uncommon, these fractures should be assessed routinely after giving anesthesia and before application of incision to choose appropriate incision and ligament reconstruction as well. The lateral incision is more appropriate for capitellum fractures with LCL injury diagnosed intra-operatively rather than an anteromedial approach.

The lateral approach of the elbow joint is a frequently used approach for fixation of capitellum fractures which provide good exposure of the whole elbow joint by releasing the extensor muscles form the lateral supracondylar ridge.^3,5,7–9^ Meanwhile, through the lateral approach it may be difficult to accurately reduce the fracture fragment and put the Herbert screw perpendicular to the fracture site especially when capitellum fracture has extended more medially to involve the trochlea. Dubberley et al mentioned that splitting of the flexor-pronator mass could have been done to assist the reduction of fracture fragment while exposing the fracture from the lateral approach.^3^ On the other hand, the anterolateral approach has the privilege of not only directly assessment of fracture site involving both capitellum and trochlea to facilitate the reduction and fixation, but also avoidance of the release of common extensor origin to prevent the postoperative extensor lag.^10–12^ However it is not free from complications with an increased chance of injury to posterior interosseous nerve. In a series of Vaishya et al, there was one case of postoperative PIN palsy with an anterolateral approach which improved completely after sometimes.^12^ So,the preference of suitable approach for capitellum fracture depends on the fracture pattern, extension of fracture, associated with other osteo-ligamentous structures, and even the experience of the surgeon. A good choice of approach gives better functional outcomes. In our series, the majority of fractures were treated by lateral approach except for five fractures with trochlear extension that were managed by the anterolateral approach.

In the present study, the average MEPI score at the latest follow-up was 90.22±8.65 (range 70 to 100) with a mean follow-up duration of 37.45±9.43 weeks (range 22 to 58 weeks). Likewise, the mean flexion and extension range was 138.41±8.22 degrees while the mean supination and pronation range was 161.59±6.79 degrees. No single case of secondary osteoarthritis and avascular necrosis of capitellum was noted in this study. Considering these clinical results, functional outcomes in our study are excellent. These results were similar to those previously published studies.^2,5,12,19^ All of these can be easily achieved by the use of Herbert screw fixation either lateral or anterolateral approach to the elbow joint.

## CONCLUSIONS

Coronal shear fractures involving the capitellum are relatively rare injuries. It is sometimes not uncommon to miss the undisplaced or minimally displaced capitellum fractures by orthopedic surgeons unless performed true lateral view of the elbow or meticulous observation and high index of suspicion was done. Careful preoperative assessment and radiological evaluation, protection of soft tissue attachment around the capitellum during surgery, appropriate surgical approach either lateral or anterolateral, anatomical reduction, and stable fixation with Herbert compression screws maintaining the minimal damage to the articular cartilage can maximize the functional outcomes and minimize the incidence of complications.

## Conflict of Interest

**None.**
